# Current Status of Cognitive Remediation for Psychiatric Disorders: A Review

**DOI:** 10.3389/fpsyt.2018.00461

**Published:** 2018-10-01

**Authors:** Eun Jin Kim, Yong-Chun Bahk, Hyeonju Oh, Won-Hye Lee, Jong-Sun Lee, Kee-Hong Choi

**Affiliations:** ^1^Department of Psychology, Korea University, Seoul, South Korea; ^2^Department of Clinical Psychology, National Center for Mental Health, Seoul, South Korea; ^3^Department of Psychology, Kangwon National University, Chuncheon, South Korea

**Keywords:** cognitive remediation, schizophrenia, mood disorders, substance use disorder, autism spectrum disorders, eating disorders, attention deficit-hyperactivity disorder

## Abstract

Cognition is an important factor that affects daily functioning and quality of life. Impairment in cognitive function is a common symptom present in various psychological disorders, which hinders patients from functioning normally. Given that cognitive impairment has devastating effects, enhancing this in patients should lead to improvements in compromised quality of life and functioning, including vocational functioning. Over the past 50 years, several attempts have been made to improve impaired cognition, and empirical evidence for cognitive remediation (CR) has accumulated that supports its efficacy for treating schizophrenia. More recently, CR has been successfully applied in the treatment of depressive disorders, bipolar disorders, attention deficit/hyperactivity disorder, and anorexia nervosa. This study critically reviews recent CR studies and suggests their future direction. This study aimed to provide a modern definition of CR, and examine the current status of empirical evidence and representative CR programs that are widely used around the world.

## Cognitive function as a target in cognitive remediation

Cognition is a collection of diverse abilities that allow an individual to recognize, process, and respond to given information. Cognitive function is involved in daily activities, such as talking to a friend or seeking a job. As people use their cognitive ability to engage in everyday life, cognitive function is considered an important factor for individuals. While cognition can be sub-divided into multiple domains (1, 2), each scholar has a different way of classifying it, and so far, there is no single taxonomy upon which all agree. In DSM-5, cognitive functions are divided into six key domains: complex attention, executive function, learning and memory, language, perceptual-motor, and social cognition. Each cognitive domain consists of more granular cognitive types (Table 1).

**Table 1:** Main cognitive domains.

**Cognitive domains**	**Subdomains**	**Assessment tools**
Attention	Basic attention span Sustained attention Divided attention Selective attention Processing speed	Digit Span Forward, Visual Span Forward Continuous Performance Test, Trail Making Test - A & B Stroop Test–word-color interference Coding, Symbol Digit Modalities Test
Executive function	Planning and decision making Working memory Responding to feedback Inhibition Flexibility	Wechsler Adult Intelligence Scale-IV (WAIS-IV): Arithmetic Wisconsin Card Sorting Test Tower of London Test Trail Making Test B Verbal Fluency test
Learning and memory	Immediate memory Recent memory: free recall, cued recall, recognition memory Semantic and autobiographical long-term memory Implicit learning Spatial memory	Rey Auditory Verbal Learning Test Wechsler Memory Scale-Revised (WMS-R): Logical memory I, logical memory II WMS-R Spatial Span Rey Complex Figure Test
Language^*^	Expressive language Receptive language	Boston Naming Test WAIS-IV Vocabulary Peabody Picture Vocabulary Test (4th ed.)
Perceptual-motor	Visual perception Visuo-constructional reasoning Perceptual-motor coordination	WAIS-IV Cancellation Rey-Osterrieth Complex Figure Test Gestalt Completion Tests Benton Neuropsychological Assessment–Facial Recognition Test Line Orientation Test
Social cognition	Recognition of emotions Theory of mind Insight Attributional style	Clinician Social Cognition Profile Social Cognitive Deficits Penn Emotional Acuity Test Mayer-Salovey-Caruso Emotional Intelligence Test: Managing Emotions

* *Language is not a targeted in cognitive remediation mainly due to its resistance to change*.

Complex attention consists of processing speed, the ability to sustain attention constantly over a period of time, the ability to selectively maintain attention to a particular stimulus among various stimuli, the ability to shift attention from one stimulus to another, and the ability to pay attention to multiple stimuli at the same time (3, 4). Attention is one of the basic cognitive functions used in everyday life. This ability allows individuals to recall a story they have just heard, to focus on a lecture, and to accept and maintain diverse and complex information. The components of executive function are planning, decision making, working memory, responding to feedback, inhibition, and flexibility (3, 5). This function allows individuals to plan, perform, and solve complex tasks in school, work, or everyday life. Learning and memory is a process of recalling new information after it has been encoded and stored. The way in which memory is categorized varies according to the academic viewpoint. Memory can be divided into immediate, recent, and remote memory. Recent memory refers to the ability to learn new information and can be further divided into verbal and visual memory (6). Memory plays a very important role in relation to people's everyday functioning. Impaired memory may cause problems in many areas, from everyday life to school or work, such as not remembering conversations, repeating the same story over and over, or not remembering appointments. The language domain can be divided into expressive and receptive language, and includes capabilities such as naming, word searching, grammar, and comprehension (3). Naming a specific object after looking at it or speaking fluently without making grammatical errors are related to language function. Perceptual-motor function consists of visual perception, visuospatial-construction, perceptual-motor coordination, and performance (3). This ability is involved in finding one's way, making things, or using tools. When the perceptual-motor domain is damaged, it is difficult to carry out activities that require hand-eye coordination such as driving a car, and it is difficult to find one's way in a familiar place. Finally, the subtype of social cognition includes recognition of emotions, theory of mind, empathy, attributional style, and insight. Social cognitive function allows individuals to behave in accordance with social norms and is involved in identifying the attitudes of others or their intentions. It is also related to vocational and social functioning and interpersonal relationships (7).

As such, cognitive function plays a very important role in daily life. Thus, cognitive deficits affect a wide range of areas such as daily life, and academic, vocational, and interpersonal areas (8–13). Impairment in cognitive function is known to be a pervasive feature of various mental disorders (14). Cognitive deficits could be one of several symptoms of psychiatric disorders, or could involve continuous change caused by one's illness (4).

## Cognitive impairment in psychiatric disorders

Cognitive impairment manifests in various psychiatric disorders such as schizophrenia, bipolar disorder, depressive disorder, attention deficit/hyperactivity disorder (ADHD), post-traumatic stress disorder (PTSD), and obsessive-compulsive disorder (OCD) (4).

Schizophrenia is a mental illness that typically characterized by cognitive decline. Many researchers and clinicians consider cognitive impairments to be core clinical features of schizophrenia and related to psychosocial functions (12, 15–18). Cognitive impairments in schizophrenia are widespread in most cognitive domains, and declines in attention, processing speed, memory, executive function, language, and social cognitive function can be observed (19–24).

In bipolar disorder, cognitive decline is associated with mood episodes (25, 26). Cognitive impairment is widespread in bipolar disorder but is of lower severity compared to schizophrenia (25).

People with major depressive disorder (MDD) experience a decline in cognitive functions such as attention, learning and memory, processing speed, and executive function (27–29). Declined cognitive function is known to predict non-response to treatment of depressive disorder and functional impairment, and is related to lower quality of life (30–32). Moreover, it has been suggested that cognitive function continues to decline, not only during the depressive episode, but also in euthymic states (33).

While ADHD is characterized by attention impairment, declines in working memory, executive function, and processing speed ability are also manifested (34–36). Regarding PTSD, it is known that there is a decline in attention, working memory, and processing speed ability (37–41).

Several studies have found that patients with OCD had lower levels of executive function and memory ability (42–47). Such impairment of cognitive function can be considered a feature that exacerbates disease symptoms in OCD (46).

Although cognitive deficits are not a prominent feature of anorexia nervosa, set-shifting and processing bias toward detail or local information are where patients experience difficulties (48, 49). Such impairments are known to be risk factors for development of the illness (50, 51) as they make patients extremely attentive to details and hinder their ability to shift attention from one task to another. Thus, previous studies show that cognitive decline is common in various mental illnesses and multiple cognitive domains are impaired.

## Cognitive remediation

Regardless of the type of mental illness, cognitive impairment affects patients' daily life and quality of life, as well as impairing the effectiveness of therapy (12, 32). In patients with schizophrenia, reduced cognitive function negatively affects quality of life and is associated with decreases in vocational and social function (1, 12, 13, 22). In depressive disorders, impaired cognitive function is known as a strong predictor of relapse of depressive episodes (52, 53). In addition, it is one of the factors that decreases work productivity in patients with depressive disorder (54). As such, cognitive impairment is not just a key symptom of mental illness, it also affects various functional areas. Hence, there has been growing interest in therapeutic methods for improving cognitive function over the past 20 years, including CR (55).

CR has been defined and updated by the Cognitive Remediation Expert Working Group (CREW) in 2005 and 2012: “Cognitive remediation is an intervention targeting cognitive deficit (attention, memory, executive function, social cognition, or meta cognition) using scientific principles of learning with the ultimate goal of improving functional outcomes. Its effectiveness is enhanced when provided in a context (formal or informal) that provides support and opportunity for improving everyday functioning.” Such therapy targets those who experience cognitive decline and aims to help individuals to function better in everyday situations such as school, work, or social situations through improved cognitive function (56, 57). Originally, CR was designed and developed to treat patients with brain lesions (58). CR was first attempted in 1915 as an attempt to improve cognitive deterioration caused by traumatic brain injury (59), and subsequently in other mental illnesses that involve cognitive decline. Since then, interest in the effectiveness of CR has been steadily increasing and has been studied continuously (60). As a result, various types of CR therapies have been developed and can be divided into compensatory and restorative approaches depending on the intervention. Compensatory approaches are designed to assist with managing cognitive decline by acquiring new skills or changing the environment, aiming to improve behavioral adaptations (61–63). On the other hand, restorative approaches aim to restore cognitive function through repetitive practice based on brain plasticity (61, 63). Additionally, CR uses a variety of learning strategies, such as errorless learning (64), reinforcement, and massed learning (65), and these strategies are applied differently depending on the type of intervention. While early researchers made improving cognitive function the primary goal of CR, later researchers dealt with the development of complementary strategies for impaired cognitive function. In addition, recent researchers have focused on generalizing the goal of CR, not just on improving cognitive function, but on improving everyday functioning (66–68).

CR is provided in a variety of ways, depending on the therapist, the patient, the treatment goals, and the program format. Early CR programs used paper and pencil for training, but recently, computer assisted cognitive training programs have been developed and utilized: PSSCogRehab2012, Cogpack, Cogmed, Lumosity and so on. As the training program is computerized, it has become easier to provide tailored training to individual patients and accessibility has also been improved. CR can be structured in a way that focuses on a specific cognitive domain or encompasses multiple cognitive domains according to treatment goals. For example, among the various cognitive domains, visuospatial function can be trained alone for 18 sessions (69) or comprehensive cognitive functions including not only neurocognitive but also social cognitive functions can be trained (70). In addition, treatment effects were reported to be greater when CR was provided with other psychosocial rehabilitation programs (57). CR is provided on a one-to-one or group basis, and the number of training sessions varies from program to program. In a study by Jang and Kim (71), a CR program was provided for 18 sessions, and in other studies, training continued for 28 sessions (72) or more than 1 year (73). According to a meta-analysis on the effectiveness of CR in patients with schizophrenia, training was carried out for an average of 12.8 weeks (74).

Currently, various CR training programs are being used in community and hospital settings. One of the comprehensive CR is the Integrated Psychological Therapy (IPT), which is a group therapy program that combines CR and social skills training for patients with schizophrenia, and a group usually consists of 5–8 people (75). IPT consists of five subprograms, which must be progressed through according to the level of complexity and can be progressed through individually if necessary. The subprograms start with cognitive differentiation and social cognition, followed by verbal communication, social skills, and interpersonal problem-solving skills. IPT is conducted two to three times a week and each session lasts 30–90 min depending on the subprogram. Another representative CR approach is the Neuropsychological Educational Approach to Cognitive Remediation (NEAR). NEAR is a cognitive rehabilitation program developed by Alice Medalia (76) for psychiatric patients. NEAR utilizes diverse learning principles to improve learning skills. To improve participants' motivation and to improve their decreased neurocognitive function, NEAR consists of several subprograms, the majority of which are computer-based. NEAR is conducted two to three times a week, with 45 min to an hour for each session with a group of six to ten people. Two of the three sessions are on cognitive function activities, and one session is a group discussion on how social skills and training programs can be applied to everyday life. Participants can choose the training program they want at each session, and their session contents differ depending on whether the cognitive function to train is basic or complex. During the training session, the therapist provides program guidance, monitors the performance of participants, and allows participants to have a more positive learning experience when they need help. NEAR is maximized when it is combined with other psychotherapy programs.

## Current status of cognitive remediation trials

CR in psychiatric disorders has been widely used in the treatment of schizophrenia where cognitive impairment is prominent, and the effectiveness of the therapy has been verified in various studies (61, 77). Recently, CR has begun to be applied to other psychiatric disorders such as bipolar disorder, depressive disorder, or attention deficit/hyperactivity disorder (76). In addition, studies using CR as a treatment for anorexia nervosa, in which cognitive decline is not prominent, are underway (78–80). Since research on CR in mental illnesses other than schizophrenia has begun relatively recently, its role and therapeutic effects are still being tested, and related studies are continuing. Proving the multifaceted application of CR, Keshavan et al. (81) have reviewed cognitive remediation in psychiatric disorders. In this study, we will focus on updating a review of recent randomized controlled trial (RCT) studies and meta-analyses that were conducted after the publication of Keshavan et al. (81). Multiple searches, until May 2018, were conducted using PubMed. The following terms were used as either key terms or keywords in the search: (“cognitive” or “cognition”) AND (“enhancement” or “training” or “remediation” or “rehabilitation”) AND (“schizophrenia” or “depression” or “depressive disorder” or major depressive disorder” or “bipolar disorder” or “attention deficit-hyperactivity disorder” or “ADHD” or “attention disorder” or “substance disorder” or “alcohol user” or “opioid user” or “methamphetamine user” or “autism spectrum disorder” or “anorexia nervosa” or “eating disorder” or “anxiety disorder”) AND (“meta-analysis” or “random” or “randomized control trial”). Among the search results, meta-analyses and randomized controlled trials (RCT) that were not reviewed by Keshavan et al. (81). were included in the current study.

### Cognitive remediation for schizophrenia

Several meta-analyses on CR have been conducted (Table 3). The earliest study by Pilling et al. (82) analyzed the effects of CR on the cognitive function of schizophrenia patients with cognitive impairment in five RCT studies. CR therapy was not effective in improving attention, verbal memory, visual memory, planning, and cognitive flexibility; thus, Pilling et al. concluded that CR is not suitable for clinical use. However, it should be noted that only six RCT studies (four studies of verbal memory and two studies in other non-cognitive domains) were analyzed in this paper and care should be taken when referring to the conclusion. Moreover, most subsequent meta-analyses agreed that CR is effective in improving cognitive function [e.g., (57, 61, 74)].

Table 3 provides a meta-analysis of the effects of the CR program on schizophrenia. In 2001, Kurtz et al. (61) conducted a meta-analysis on 11 RCT studies (*n* = 181) that utilized a rehabilitation strategy program to improve Wisconsin card test performance. Among the neurocognitive functions that were explored in the meta-analysis, improvements were found in executive function, attention, and memory, and the effect size was 0.98. Subsequently, Wykes et al. (57) conducted a meta-analysis of 40 RCT studies (*n* = 2104) and found a moderate effect size for overall cognitive function (*d* = 0.45). Most meta-analyses have shown that CR is effective in improving cognitive function in patients with schizophrenia (57, 59, 61, 74, 77). However, except for a study by Kurtz et al. (61), the effect size of CR on cognitive function has been found to be moderate in most studies. There are several possible explanations for the effect size not being large. First, there is noticeable diversity of CR between studies. Although Wykes et al. (57) noted they did not find a significant difference in various CR approaches, nonetheless, they found that drill and strategy coaching produced a greater effect than drill and practice. This finding is consistent with the study of McGurk et al. (74), who reported that providing CR within the context of larger psychosocial rehabilitation is associated with a better outcome than only providing CR (57). The various components of CR should be considered for meta-analyses, including whether it is provided individually or in a group, and with a computerized software or paper and pencil; however, most studies have not considered this or mixed them (57, 59, 74, 77). Second, in most meta-analyses, the distinction between cognitive domains as outcomes targeted by CR versus those not targeted is not clear (57, 59, 61, 74). Grynszpan et al. (77) suggested this moderate effect size might have resulted from the mixed use of cognitive domains targeted and not targeted by CR. Third, most studies did not consider the amount of time required and intensity of training (59, 61, 77). McGurk et al. (74) found that hours of training was associated with effectiveness of CR. Given that the hours of training in most CR RCT studies is <30 h, there is much difference in the training time between trials and in practice. Therefore, the moderate effect size of CR may not be disappointing.

CR not only enhances cognitive function but also promotes changes in psychiatric and psychosocial symptoms, and a number of related meta-analyses have been performed. Most of the meta-analyses on CR, except for the early Pilling et al. (82) study, reported a reduction in psychiatric symptoms (59, 74, 83, 84). The effect sizes were small to moderate (*d* = 0.19 – 0.52), and among the other studies, a CR meta-analysis concerning early psychosis showed the smallest effect size. In early psychosis, baseline function is less impaired compared to other kinds of schizophrenia (85), resulting in low effectiveness. The most recent meta-analysis by Cella et al. (83) reported the effects of CR on negative symptoms (*g* = −0.30). While positive symptoms can be alleviated by drugs, drugs to treat negative symptoms are not yet available. Therefore, this result suggests the possibility of using CR as a treatment to reduce negative symptoms as well as improving neurocognitive function. Along with change in psychiatric symptoms, CR training is effective in improving psychosocial functions such as in daily life, and in vocational and social functioning (57, 75). The effect sizes of improvements in psychosocial function ranged from 0.18 to 0.51, and in a meta-analysis conducted by Chan et al. (86), CR increased the employment rate by 20%, the annual workday by 19.5 days, and the annual salary by $959. Considering the wide effects of CR on daily life, study, work, and interpersonal relationships (12, 13, 87), improvement of psychosocial function through CR can be an important factor in returning patients with schizophrenia to the community.

In addition to the modest efficacy of CR in schizophrenia that Keshavan et al. (81) have addressed, as early intervention is being highlighted, more studies on CR are being carried out focusing on early psychosis patients. Revell et al. (84) analyzed 11 RCT studies on CR with early psychosis patients. Unlike the majority of meta-analyses that agreed that CR is effective in improving cognitive function, Revell et al. (84) reported no significant improvement in neurocognitive function (*d* = 0.13). In addition, reduction of psychiatric symptoms and improvement of function were smaller (*d* = 0.19, *d* = 0.18). The authors noted that these results resemble those of the meta-analysis conducted by Wykes et al. (57), although the effect size was small. The authors further noted that the small effect size was due to higher functional levels, including cognitive function, in early psychosis than those in chronic schizophrenia. This may result in chronic schizophrenia patients experiencing larger effects from CR. Since the study by Revell et al. (84) is the only meta-analysis on CR with early psychosis, more research is needed to support or refute the results of this study. Although the majority of studies have been conducted in the United States or European countries, recently CR RCTs have been reported in Asian countries (88–90).

### Cognitive remediation for major depressive disorder

While decreased cognitive function in major depressive disorder (MDD) is known to correlate with treatment reactivity, psychosocial function, and relapse of depressive episodes (30–32, 52, 53, 91–93), there are only a few studies on CR with MDD patients. Unlike schizophrenia, for which there are several meta-analyses on CR effectiveness, only two meta-analyses were conducted on the effectiveness of CR on MDD. In addition to the meta-analysis on CR with affective and schizoaffective patients (55), which was reviewed by Keshavan et al. (81), a meta-analysis on the effectiveness of CR on MDD patients was conducted. Motter et al. (94) analyzed nine RCT studies on the effects of CR on global cognitive function, depressive symptom severity, and psychosocial function in MDD. The results showed that, among the neurocognitive functions, attention, working memory, and overall cognitive function were improved, whereas the effect sizes of verbal memory and executive function were not statistically significant (*g* = 0.08 and *g* = 0.20, respectively). The effects of CR on depressive symptom and psychosocial function further suggest that CR can be a treatment option for MDD. It should be noted, however, that only nine studies were included in this meta-analysis, thus the number of studies used to analyze the effect size of each domain was small. A large effect size of 1.05 was reported for global cognitive functioning, but since it was based on the results of two assessment tools used in one study (95), care should be taken in interpreting this result (94). In addition, the inclusion criteria of participants, such as cognitive impairment, were not considered in the analysis, nor was the duration of training, even though the studies showed different durations of training [6–64 sessions; (94)]. Given the diversity in effect sizes of CR, analysis of moderators would help clarify its effect on depression.

In relation to the effect of CR on depressive symptoms, in several studies included in this meta-analysis (94), participants were given CR either simultaneously with an antidepressant or another psychotherapy as part of a treatment session. Considering this, Motter et al. (94) reported that whether the alleviation of depressive symptoms was due to improvement in cognitive function or to enhancement of usual treatment could not be identified.

Trapp et al. (96), who were not included in the meta-analysis by Motter et al. (94), also studied the effect of CR on patients with MDD. 46 patients with MDD participated in this study and were randomly assigned to either a control group or an experimental group, receiving usual treatment or 12 sessions of CR along with usual treatment. The experimental group showed a larger improvement in neurocognitive indexes such as executive function, working memory, and memory (*d* = 0.52–0.98). Unlike the changes in neurocognitive function, CR did not have a significant effect on the depression symptom score. The lack of a significant reduction in depressive symptom in Trapp et al. (96) contrasts with the results of the meta-analysis by Motter et al. (94) in which depressive symptoms were reduced. For these results, Trapp et al. (96) reported that the experimental group receiving CR had a lower depressive score than the control group, although this was not statistically significant. These results were presented in trials that did not control for drugs, making it difficult to conclude whether CR is effective in depressive symptoms. Further research should investigate the mechanisms of how improvement of cognitive functioning affects depressive symptoms.

In this respect, the authors further suggested that the depressive symptoms of MDD patients who participated in this study were milder than those of MDD inpatients in the UK or Australia, and thus no statistically significant change in symptom was observed.

To date, only two meta-analyses and several RCT studies on the effects of CR on MDD have been published. These studies have shown that CR is effective in improving cognitive function. However, the number of studies is insufficient to be conclusive, and there is also insufficient data on whether improved cognitive functioning is sustained, or whether improvements in cognitive functioning are translated into improvement of daily functioning and depressive symptom reductions. Therefore, more research results should be accumulated, and if CR reduces depressive symptoms, it should be proven that the changes in symptom were due to CR rather than the influence of other factors. Furthermore, it should also be verified that CR induces significant changes in other areas such as psychosocial function, relapse rates, and suicide attempts in MDD.

### Cognitive remediation for bipolar disorder

Bipolar disorder, characterized by recurrent depressive and manic episodes, has been extensively associated with neurocognitive impairment and low levels of performance in attention, memory, working memory, executive function (26), and social cognition domains (97, 98). Such deterioration of cognitive function interferes with everyday functioning (99), quality of life (100), psychosocial activities, and productivity in bipolar patients (54). Currently there is no drug or psychotherapy that has proven effective in improving impaired cognitive function in bipolar disorder (101). CR outcome studies with bipolar disorder are scarce, and a few RCT studies have been conducted. In the non-RCT studies that targeted verbal memory, attention, and executive function of patients with bipolar disorder, they found improved targeted cognitive functions after CR (102–104). However, since participant numbers were low, and these studies were not conducted as RCTs, these results should be considered as preliminary studies only.

Only two RCT studies on bipolar disorder have been reported. Torrent et al. (105) conducted an efficacy trial of functional remediation programs, including various training such as psychoeducation, cognitive training, communication, and interpersonal skills training. They reported that psychosocial functioning (e.g., leisure, interpersonal, cognitive domains) in the functional remediation group improved compared to the control group. However, in this study, the neurocognitive assessments were not employed to measure cognitive functioning. Demant et al. (101) conducted CR in patients with bipolar disorder and reported null effects of CR compared to the control group. However, it should be noted that the study sample size was small, and it is unclear whether study participants has neurocognitive impairments at baseline, as they did not measure objective neurocognitive impairments when recruiting and screening participants. These methodological issues made results inconclusive. Therefore, to investigate the effect of CR on neurocognitive and psychosocial function in bipolar disorder, more research utilizing various methods (e.g., objective neurocognitive and functional assessments) in a larger sample with cognitive impairments should be conducted.

### Cognitive remediation for attention deficit-hyperactivity disorder

Attention deficit/hyperactivity disorder (ADHD) is characterized by ongoing carelessness and impulsivity-hyperactivity patterns that start from childhood. Therapeutic methods combining drug therapy and psychotherapy are recommended, and pharmacotherapy has been used as a priority treatment since the short/mid-term effectiveness of drugs used for ADHD has been confirmed. However, pharmacotherapy is not equally effective in all ADHD patients (106), and the expected effects of long-term use remain unclear (107).

In recent years, CR has been studied as a potential treatment for ADHD. It has been argued that CR for impaired neurocognitive function, which is expected to mediate the pathophysiology of ADHD, will result in decreased symptoms of ADHD and improved functioning (108). As CR has become a potential treatment option, a number of CR studies have been conducted on ADHD. Keshavan et al. (81) reviewed studies to introduce how CR is done with ADHD and highlighted its usability with results of improvement in ADHD symptoms and other neurocognitive domains such as working memory, attention, and cognitive flexibility. Since this review was published, meta-analyses on the effectiveness of CR on ADHD have also been published and more RCT studies been conducted. Sonuga-Barke et al. (109) examined the effects of CR training on symptom severity of ADHD in six RCT studies. The results showed that there was a significant change in the overall symptoms of ADHD [standardized mean differences [SMD] = 0.64]. However, this resulted from not using blinded measures when assessing ADHD symptoms, and the effect of CR on ADHD symptoms was reduced when using a probability blinded measure and was not statistically significant (SMD = 0.23).

Differences between using and not using a blinded measure were replicated in two later meta-analyses. A meta-analysis by Rapport et al. (110) also reported similar results to the study by Sonuga-Barke et al. (109) and reported that among neurocognitive functions, short-term memory improved after having short-term memory training (*d* = 0.63). Comparing these two meta-analyses, Rapport et al. (110) included two additional RCT studies as well as several non-RCT studies to increase statistical power. In the results, there was difficulty in interpreting the effects of CR on ADHD symptoms and impaired neurocognitive function reported by Rapport et al. (108, 110). In a recent meta-analysis by Cortese et al. (108), 16 RCT studies examining the effects of cognitive remediation training on ADHD symptoms, neurocognitive functions, and academic skills were reviewed. Based on the analysis, CR had significant effects on laboratory tests of working memory and parent ratings of executive function (verbal working memory: SMD = 0.52; visual working memory: SMD = 0.47; executive function: SMD = 0.35), and further had effects on ADHD symptom severity and inattentive symptom (SMD = 0.37; SMD = 0.47). However, as with other meta-analyses, the use of a blinded measure reduced the level of change observed in ADHD symptom severity and inattentive symptoms (SMD = 0.20, SMD = 0.32). Furthermore, there was no significant change in the academic ability of ADHD patients.

Six RCT studies were conducted after these three meta-analyses were published. While five studies reported improvement in the working memory domain of the group that had received CR (111–115), Bigorra et al. (116) reported no significant change in their study. Only Bigorra et al. (114) reported improvement in ADHD symptoms, while the other five RCT studies did not investigate or report significant change in ADHD symptoms and academic skills (111–113, 115, 116).

The effectiveness of CR training is limited when the related symptoms are assessed using blinded measures, contrary to what was discussed regarding reducing the symptoms of ADHD and improving functions. A meta-analysis showed improvement in working memory, whereas no significant change was observed in ADHD symptoms. In a CR program, working memory training was effective only in improving working memory, and had no effect on other neurocognitive processing functions, and there was no evidence that the effects of training generalized to other important daily functions (108). Such a conclusion is further supported by RCT studies conducted since 2015, where improvement was only found in working memory. Thus, unlike what has been discussed so far, training of impaired neurocognitive function that is expected to mediate the pathophysiology of ADHD does not affect the symptoms and function of the disease. Therefore, the effects of CR should be devised not only to improve the cognitive function being trained, but also to generalize the effects of training to other neurocognitive functions, symptoms of disease, and psychosocial functions.

### Cognitive remediation for substance use disorders

Impairments in various cognitive domains such as working memory, executive function, and attention are also associated with substance use disorders (SUD) and such impairments further hinder treatment adherence and outcomes (81). Starting with studies such as applying a single dose of Go/No-go response training to assess response inhibition, substance intake, and implicit attitude toward substance inhibition (117–119), recent studies have administered CR additively to facilitate treatment and improve disorder-related functional outcomes (120).

Since the review on the effects of CR on SUD by Keshavan et al. (81), more RCT studies have been conducted. While the participant characteristics varied from alcohol users to methamphetamine or opioid users, six studies have applied CR in substance users (100, 121–125). Among these, five studies measuring cognitive function reported improvement in neurocognitive outcomes (100, 121–123, 125). All these studies showed improvement in working memory, while the study conducted by Rass et al. (122) did not show improvement in dissimilar working memory measures. In addition to working memory, improvements in executive function, learning, and processing speed were also found (100, 122, 124). Substance use related outcomes have also been reported. Use of methadone was stably maintained in the CR group compared to the active control group where substance intake increased (122, 125). However, Bell et al. (123) reported no between-group difference in days of abstinence. Furthermore, improvements in psychosocial outcomes such as depressive symptom, desire for drugs, and self-regulation were also observed (124).

Considering these recent RCT studies, CR seems to be a promising approach in treating substance use disorders and its effectiveness has been widely studied. Since Keshavan et al.'s (81) review, several RCT studies have shown that CR is effective for SUD patients without cognitive impairments. This suggests that CR would be applicable in SUD individuals who do not have cognitive impairments and requires further research to investigate the relationship between mechanisms of cognitive function enhancement and other outcomes.

### Cognitive remediation for autism spectrum disorder

Autism spectrum disorder (ASD) is characterized by impairment in social interaction, communication, and interpersonal relationships, and restricted and repetitive behavior and interests (3). These deficits interfere with daily life and may cause impairments in psychosocial functions such as social or vocational functions (3). People with ASD are also known to experience impairments in cognitive domains such as executive function or memory (126–128). Hence, utilizing CR as a treatment option for ASD was considered as beneficial, leading to studies being conducted on this topic (129). Keshavan et al. (81) reviewed three studies related to CR on ASD and commented that these studies were very limited. Since Keshavan et al.'s (81) review, two RCT studies were conducted for the first time.

The first RCT study on CR effects on ASD was conducted by Vries et al. (129) and reported that no significant change was shown in neurocognitive and psychosocial function outcomes and in disorder-related symptoms. Only a trend level of improvement in working memory, cognitive flexibility, and ADHD behavior was shown in this study. In this study, cognitive impairment was not considered in the inclusion criteria. By including participants who did not have cognitive impairments in CR, even excluding motivational issues, this could underestimate the effect of CR. Miyajima et al. (130) reported improvements in working memory, verbal fluency, and planning; however, such results should be interpreted carefully as the sample size in this study was only 14. Since only two RCT studies have been conducted on this topic, it is hard to conclude whether CR is a promising treatment for ASD. Just as Keshavan et al. (81) said, the field is still in its infancy and more studies on this topic should be conducted to investigate the effectiveness of CR on ASD.

### Cognitive remediation for anorexia nervosa

CR is also used in the treatment of anorexia nervosa, where cognitive decline is not prominent. Patients with anorexia nervosa do not experience severe global cognitive impairment, like in schizophrenia patients, but cognitive inflexibility and processing bias toward detail or local information are characterized, and these factors contribute to disease intensification and delay patient recovery (131–135). In patients with anorexia nervosa, CR is added to the usual treatment for the purpose of improving cognitive flexibility and central integration ability, not improving cognitive function. However, since CR has been applied just recently to anorexia nervosa, the purpose of CR differs for each study. Most studies use CR to improve the effectiveness of treatment for anorexia nervosa and to reduce treatment drop-out rates. The CR program for anorexia nervosa was developed by Tchanturia in 2010 (136) and places greater emphasis on the process of thinking than other CR programs.

There have been four RCT studies on the effectiveness of CR for anorexia nervosa, and different areas were measured for each study. There was no significant difference between the groups in the study by Lock et al. (78) and Dingemans et al. (79). However, in Brockmeyer et al. (137), the cognitive remediation group performed better in cognitive set-shifting than the control group. Since cognitive set-shifting is a form of cognitive flexibility, this demonstrates significant change in the target area of the CR program. For changes in body weight, Lock et al. (78) and Dingemans et al. (79) reported no significant differences between groups. Furthermore, Stienglass et al. (138) reported that calorie intake was lower in the cognitive rehabilitation group than in the control group. Dingemans et al. (79) reported a significant improvement in the CR group compared to the control group, considering the quality of life of patients with anorexia nervosa. Taking these results together, the effects of CR for the anorexia nervosa on cognitive flexibility have not been consistent. In addition, it is unknown whether CR is effective for anorexia nervosa because the areas searched for each study were different and mixed results were derived. In addition, considering that this field is still in its infancy with a low number of RCT studies, it might be premature to discuss the effectiveness of CR in anorexia nervosa at present.

### Attention training for social anxiety disorder

Attention bias modification (ABM) is a cognitive re-training program of the implicit attention biases that are known to be a causal information processing factor resulting in anxiety symptoms. Individuals in ABM are repetitively trained to shift their attention from negative to either neutral or positive stimuli (e.g., disgusted face to happy face) on a computer screen delivered either in the clinic or online at home. This intends to implicitly change negatively biased to more positively biased thought habits, thereby reducing anxiety symptoms. Since the first study of ABM (139), two meta-analyses of RCTs have looked at the efficacy of ABM on the change of attention bias and anxiety symptoms. The first included 15 RCTs of ABM and showed small, but significant effects in attention bias (*g* = 0.30), social anxiety symptoms (*g* = 0.27), and stress to speech challenges [*g* = 0.46; (140)]. The second study focused on 11 RCTs, especially in clinically diagnosed anxious patients, and found a medium effect on the change of attention bias (*d* = 0.61) and a small effect on anxiety symptoms, as rated by clinicians [*d* = 0.42; (141)]. In moderation analyses, both studies found that ABM delivered in the clinic or laboratory produced larger effect sizes than those delivered online. Following these meta-analyses, another RCT on ABM in those diagnosed with social anxiety disorder showed medium to large effects on both clinician ratings (*ds* = 0.57 ~ 0.70) and self-reports of social anxiety symptoms [*ds* = 0.70 ~ 0.85; (142)].

Beyond behavioral change mechanisms, neural working processes of cognitive functions underlying anxiety disorders following ABM are also important as it provides insight into the therapeutic mechanism underlying CR in attention bias. Several studies have been conducted to investigate brain change mechanisms in attention bias by combining CR with functional magnetic resonance imaging or transcranial direct current stimulation (tDCS). Two previous studies found that CR training decreased amygdala activation when used to target the avoidance of a threat stimulus (143) and increased activation of the lateral prefrontal cortex in response to emotional stimuli (144). Using ABM combined with either active anodal tDCS targeting left dorsolateral prefrontal cortex (DLPFC) or sham tDCS, Clarke et al. (145) found a significant change in attention bias in the active tDCS condition relative to the sham tDCS condition. Heeren et al. (146) also reported consistent results showing that active anodal tDCS targeting the DLPFC showed a significant reduction in time at the gaze remained fixed on the threat compared to their counterpart (ABM + sham tDCS). Recently, Heeren et al. (147) showed that anodal tDCS per se (without ABM) targeting left DLPFC can decrease attentional bias for threat of individuals with social anxiety disorder. This study demonstrated the possibility that tDCS could be used for cognitive interventions as well as improving the effectiveness of cognitive intervention in anxiety disorders (147) and other psychiatric disorders (148–150).

It is assumed that anxiety symptoms are triggered by a dysregulated attention to threat; anxious individuals habitually attend to negative instead of positive stimuli. This tendency might be accounted for by the accumulating evidence on brain studies showing that the amygdala is more activated, whereas the lateral PFC is less activated in response to a threat stimulus, suggesting imbalance between the top-down (attentional control) and bottom-up (emotional processing) processes (151). Although two meta-analyses on ABM targeting the modification of biased attention showed small effects on attention biases and anxious symptoms, it is promising that there is evidence indicating neural change mechanisms following ABM. Further ABM studies, combined with neuromodulation techniques such as tDCS, are warranted to focus on how its efficacy can be increased for its clinical utility.

## Discussion

CR has been conducted in various psychiatric disorders, and a number of studies have suggested that CR is effective in improving impaired cognitive functioning. However, conducting CR in psychiatric disorders other than schizophrenia is a relatively recent phenomenon, and not enough studies have been accumulated yet.

Several CR studies have shown that CR can be used for other purposes (e.g., relief of symptoms, enhancement of psychosocial functioning) as well as improvement cognitive functioning. These additional effects of CR were limited compared to the improvement of cognitive functioning, which may mean that cognitive deficit in most psychopathologies represent the effects, rather than the causes of psychopathology. Nevertheless, It is important that CR can affect other outcome variables besides cognitive function, and it implies the future possibility of CR. It is important to clearly understand the mechanism by which cognitive enhancement leads to other changes. What changes does cognitive functioning lead to? Which cognitive functioning is critical to induce the changes? Are these changes maintained? Is CR more effective (or more efficient) than other treatments? These questions will help to better understand the process by which CR leads to other changes. In future CR studies, it is important to investigate what occurs beyond merely improving specific cognitive functioning in CR. A cognitive test known to measure specific cognitive domains often requires the use of multiple cognitive domains simultaneously. In this case, what does it mean when one specific cognitive domain is improved and another is not? It is important to classify cognitive domains in both the study of CR and in practice. However, research of CR needs to go a step further. We suggest that beyond the demonstration of specific cognitive function enhancement in CR studies, it is important for future research to investigate the consequences of specific enhancement of cognitive functioning on the overall clinical/emotional symptoms of the psychological disorder, through CR.

We discussed the methodological issues that some results of CR studies have been inconsistent, and the effect size of treatment differed from study to study (see Tables 2–8). Among them, we noticed that a number of CR studies have used various independent CR programs, with diversity in duration, intensity, structure, software, target cognitive domain, measurement tools, and purpose of treatment. In terms of research, this diversity may lead to inconsistent results. Thus, there is a need to establish standard procedures for CR trials for research, or it is necessary to agree on what is the most essential element of CR.

**Table 2:** Meta-analysis of cognitive remediation (CR) effect on schizophrenia.

**References**	**Number of included studies and participants (N)**	**Participants characteristic**	**Assessed domains**	**Types of cognitive remediation program**	**Moderator**	**Neurocognitive outcomes (effect size)**	**Psychiatric symptom outcomes (effect size)**	**Psychosocial function outcomes (effect size)**	**Country**
Pilling et al. (82)	5 studies (*N* = 170)	Schizophrenia Schizoaffective disorder	Global cognitive function Psychiatric symptom	Computerized attention training Memory training Problem-solving and complex planning training	–	No effects on neurocognitive function: Attention (*d* = 0.11) Verbal memory (*d* = 0.14) Visual memory (*d* = 0.34)	No benefit on mental state (*d* = −0.23)	–	UK, USA
Kurtz et al. (61)	11 studies (*N* = 181)	Schizophrenia	Executive function Attention Memory	Strategies to improve Wisconsin Card Sorting Test performance training	–	Enhancement of executive function (*d* = 0.98 or 0.96) Attention: mixed result Memory: non-conclusive result	–	–	Canada, The Netherlands, UK, USA
Twamley et al. (59)	17 studies (*N* = 181)	Schizophrenia	Neurocognitive function Psychiatric symptom Everyday functioning	Computerized training Non-computerized training Learning strategies utilized training Restorative approach training Compensatory approach training	–	Improvement in neurocognitive function (*d* = 0.32)	Psychiatric symptom decreased (*d* = 0.26)	Improvement in everyday functioning (*d* = 0.51)	Australia, Canada, Germany, UK, USA
McGurk et al. (74)	26 studies (*N* = 1,151)	Schizophrenia Schizophreniform Schizoaffective disorder	Global cognitive function Psychiatric symptom Psychosocial function	Computerized training Non-computerized training Cognitive remediation program with social cognition exercises	Verbal learning and memory domain: More hours of cognitive remediation compared with fewer Drill and practice compared with drill and practice plus strategy coaching Functioning Improvement domain: Adjunctive psychiatric rehabilitation compared to none Drill and practice plus strategy coaching compared to drill and practice only Older than younger	Improvement in neurocognitive function (*d* = 0.41)	Psychiatric symptom decreased (*d* = 0.28)	Improvement in psychosocial function (*d* = 0.36)	Australia, Canada, Germany, Spain, The Netherlands, Norway, UK, USA
Wykes et al. (57)	40 studies (*N* = 2,104)	Schizophrenia Schizoaffective disorder	Global cognitive function Psychiatric symptom Psychosocial function	Computerized restorative approach training Compensatory approach training 1 to 1 training Group-based training	None of variables found to be statistically significant	Improvement in global cognitive function (*d* = 0.45)	Psychiatric symptom decreased (*d* = 0.18)	Improvement in psychosocial function (*d* = 0.42)	Australia, Canada, France, Germany, Israel, Italy, Spain, The Netherlands, Norway, UK, USA
Grynszpan et al. (77)	16 studies (*N* = 805)	Schizophrenia Schizoaffective disorder	Global cognitive function	Computer-assisted cognitive remediation (CACR)	None of the moderator variables found to be statistically significant	Improvement in global cognitive function (*d* = 0.38) Improvement in social cognitive function (*d* = 0.64)	–	–	Germany, USA
Chan et al. (86)	9 studies (*N* = 740)	Schizophrenia Schizoaffective disorder	Employment rates Total days of work in a year Total annual earnings	Computer-assisted cognitive remediation (CACR)	–	–	–	20% higher employment rate, Worked 19.5 days longer in a year, Earned US$959 more in total annual earnings	Germany, Italy, Japan, Singapore, USA
Roder et al. (75)	36 studies (*N* = 1,601)	Schizophrenia	Global cognitive function Negative symptom Psychosocial function	Integrated Psychological Therapy (IPT)	None of the moderator variables found to be statistically significant	Improvement in global cognitive function (*d* = 0.53)	Psychiatric symptom decreased (*d* = 0.52)	Improvement in psychosocial function (*d* = 0.42)	Austria, Brazil, Canada, Germany, Italy, Japan, The Netherlands, Norway, Spain, Switzerland, Panama, USA
Cella et al. (83)	45 studies (*N* = 2,511)	Schizophrenia Schizoaffective disorder	Negative symptom	Computerized training and paper-pencil based training that utilize learning strategies	None of the moderator variables found to be statistically significant	–	Negative symptom decreased (*g* = −0.30)	–	Australia, Canada, Germany, India, Italy, Iran, The Netherlands, Norway, Spain, UK, USA
Revell et al. (84)	11 studies (*N* = 615)	Early psychosis	Global cognitive function Psychiatric symptom Psychosocial function	Computerized training Paper-pencil based training Learning strategies utilized training Repetitive practice Group based training	Global cognition domain: Male compared to female Functioning domain: Adjunctive psychiatric rehabilitation than none Setting domain: Group compared to one-to one	No significant improvement in neurocognitive function (*d* = 0.13)	Psychiatric symptom decreased (*d* = .19)	Improvement in psychosocial function (*d* = .18)	Australia, Denmark, Norway, Spain, Switzerland, UK, USA

*(-): Not assessed domain*.

**Table 3:** Meta-analysis and randomized control trial(RCT) studies on CR effect on major depressive disorder(MDD).

**References**	**Study type**	**Number of included studies and participants (N)**	**Participants characteristic**	**Assessed domains**	**Types of cognitive remediation program**	**Neurocognitive outcomes (effect size)**	**Depressive symptom severity (effect size)**	**Psychosocial function outcomes (effect size)**	**Country**
Motter et al. (94)	Meta-analysis	9 studies (*N* = 157)	MDD	Global cognitive function Depressive symptom severity Psychosocial function	Cognitive remediation program training global cognitive function Computerized training: PSSCogRehab, Neuropsychological Educational Approach to Cognitive Remediation(NEAR)	Improved attention (*g* = 0.67) Improved working memory (*g* = 0.72) Improved global cognitive function (*g* = 1.05)	Decreased depressive symptom severity (*g* = 0.43)	Improved psychosocial function (*g* = 0.48)	Australia, Canada, Mexico, UK, USA
Trapp et al. (96)	RCT	46 (23^*^/23)	MDD	Global cognitive function Depressive symptom severity	Cognitive training software X-Cog	Improved executive function, working memory and memory	No significant decrease in depressive symptom severity	–	Germany

(cognitive training program participants

* */control group). (-): Not assessed domain*.

**Table 4:** RCT studies on CR effect on bipolar disorder.

**References**	**Participants (N)**	**Participants characteristic**	**Assessed domain**	**Types of cognitive remediation program**	**Neurocognitive outcomes**	**Psychosocial function outcomes**	**Country**
Torrent et al. (105)	183 (77^*^/82/80)	Bipolar disorder	Overall psychosocial function	Functional remediation program addressing neurocognitive domains (attention, memory and executive function) with focus on enhancing functioning in daily routine	–	Improvement in functional outcome compared to treatment as usual group	Spain
Demant et al. (101)	40 (18^*^/22)	Bipolar disorder in partial remission	Verbal memory, Attention, Executive function, Psychosocial function	Short-term group-based computerized training	No significant effect relative to control group Verbal fluency improved at follow-up	Cognitive remediation participants improved on subjective sharpness right after the training ended and quality of life improved at follow-up	Denmark

Cognitive training program participants

* */comparative group/standard treatment group or treatment as usual group*. *(-) Not assessed domain*.

**Table 5:** Meta-analysis and RCT studies on CR effect on attention-deficit/hyperactivity disorder(ADHD).

**References**	**Study type**	**Number of included studies and participants (N)**	**Participants characteristic**	**Assessed domains**	**Types of cognitive remediation program**	**Neurocognitive outcomes (effect size)**	**Academic skills (effect size)**	**ADHD symptom severity (effect size)**	**Country**
Sonuga-Barke et al. (109)	Meta-analysis	6 studies (*N* = 249)	ADHD	ADHD symptoms	Working memory training Attention training	-	-	Significant change in overall ADHD symptoms (Standard Mean Difference [SMD] = 0.64)	Australia, Israel, Sweden, USA
Rapport et al. (110)	Meta-analysis	25 studies (*N* = 913)	ADHD	Cognitive function Academic skills Behavior	Computerized training: CogMed, Independently developed cognitive remediation treatment Training focused on attention	Training short-term memory improved short-term memory (*d* = 0.63) No significant effect after training attention and executive function	No significant change	No significant effect on behavior	Australia, Canada, Germany, Israel, The Netherlands, Spain, Sweden, UK, USA
Cortese et al. (108)	Meta-analysis	16 studies (*N* = 759)	ADHD	ADHD symptom Neurocognitive function Academic skills	Executive function training Working memory training Inhibition training	Significant effect on laboratory test of working memory (Verbal: SMD = 0.52; Visual: SMD = 0.47) Significant effects on executive function (SMD = 0.35)	No significant change	Significant effect on ADHD symptom severity and inattentive symptom (SMD = 0.37; SMD = 0.47) Working memory training had no significant effect on ADHD symptom severity	Canada, Israel, The Netherlands, Norway, Sweden, USA
van der Donk et al. (111)	RCT	100 (50^*^/50)	ADHD	Neurocognitive functions, academic performance, quality of life, behavior in class	Cogmed	Visuospatial memory improvement	No significant change	No significant change	The Netherlands
Dovis et al. (112)	RCT	89 (31^*^/28^*^/30)	ADHD	Executive function, working memory, ADHD symptom, quality of life	Training inhibition, working memory, cognitive flexibility	Improvement in visuospatial short term memory (*$\eta_{p}^{2}$* = .16 -.09), inhibition (*$\eta_{p}^{2}$* = 0.09 and 0.07)	No significant change	No significant change	The Netherlands
Mawjee et al. (113)	RCT	97 (32^*^/33^*^/32)	ADHD	Working memory, everyday functioning, academic performance, ADHD symptom	Cogmed	Improvement in working memory but no evidence of any transfer effects	No significant change	No significant change	Canada
Bigorra et al. (114)	RCT	66 (36^*^/30)	ADHD	Executive function, ADHD symptom, working memory	Cogmed Working Memory Training	Improvement in executive function in (*d'* = −0.78), working memory (*d'* = −0.61),	-	Improvement in ADHD symptoms (*d'* = −0.39 to −0.69)	Spain
Bigorra et al. (116)	RCT	66 (36^*^/30)	ADHD	Executive function: decision making, working memory, theory of mind	Cogmed Working Memory Training	No significant change	-	No significant change	Spain
Dentz et al. (115)	RCT	44 (23^*^/21)	ADHD	Working memory, executive function, ADHD symptom	Cogmed	Improvement in verbal working memory (*η_*p*_*^2^ = 0.09) and visuospatial working memory (*η_*p*_*^2^ = 0.18)	-	No significant change	Cananda

(cognitive training program participants

* */comparative group/standard treatment group or treatment as usual group)*. *(-) Not assessed domain*.

**Table 6:** RCT studies on CR effect on substance use disorders.

**References**	**Number of participants (N)**	**Participants characteristic**	**Assessed domains**	**Types of cognitive remediation program**	**Neurocognitive outcomes (effect size)**	**Substance related outcomes (effect size)**	**Country**
Gamito et al. (121)	54 (26^*^/28)	Alcohol use disorder	Attention, processing speed, global cognitive function	mHealth	Improvement in frontal lobe functions (η_p_*^2^* = 0.16)	–	Portugal
Rass et al. (122)	56 (28^*^/28)	Methadone maintenance patients	Memory, attention, executive function, reasoning, substance use and functional outcomes	Cogmed	Improvement in trained working memory measures (*p* = 0.047)	Stable use of drug while control group showed increase in use	USA
Bell et al. (100)	31 (15^*^/16)	Veterans with alcohol use disorder	Substance use outcomes, attention, processing speed, executive function, memory	Auditory and visual Posit Science software: auditory memory task, sensory processing task	Greater improvements on verbal memory and learning (*d* = 1.01; *d* = 1.09)	Not reported	USA
Bell et al. (123)	48 (24^*^/24)	Veterans with substance use disorder	Working memory, executive function, attention, processing speed, visual and verbal learning and memory, SUD outcomes	Auditory and visual Posit Science software: auditory memory task, sensory processing task	Improvement in working memory (*d* = 0.66), executive function (*d* = 0.68)	Both group showed similar days of abstinence	USA
Brooks et al. (124)	41 (20^*^/15)	Methamphetamine use disorder	Mood, impulsivity, desire for drug, self-regulation, executive function, processing speed	Working memory training	–	Better mood (*t* = 2.784), higher percentage feeling of self-control (*t* = 2.736), better self-regulation (*t* = 2.442) better impulsivity and anxiety in CR group compared to baseline	South Africa
Rezapour et al. (125)	51 (23^*^/28)	Opioid use disorder male	Attention, visuospatial process, working memory, verbal skills, executive function, depression, intensity of withdrawal symptoms, drug use, treatment retention	NEuroCognitiveRehabilitation for Disease of Addiction programme (NECOREDA): training global cognitive domains	Improvement in working memory (*η_*p*_*^2^ = 0.30), learning (*η_*p*_*^2^ = 0.16), switching (*η_*p*_*^2^ = 0.11), processing speed and memory span (*η_*p*_*^2^ = 0.10 for each)	Analyzing participants with history of amphetamine use, CR group showed lower use of amphetamine (*d* = 1.24)	Iran

(Cognitive training program participants

* /comparative group or treatment as usual group) (-) Not assessed domain

**Table 7:** RCT studies on CR effect on autism spectrum disorder.

**References**	**Participants (N)**	**Participants characteristic**	**Assessed domains**	**Types of cognitive remediation program**	**Neurocognitive outcomes**	**Psychosocial function outcomes**	**Autism spectrum disorder related symptoms**	**Country**
Vries et al. (129)	115 (40^*^/37^*^/38)	Autism spectrum disorder (ASD)	Working memory, executive function, attention, daily life function, daily life function, ADHD behavior	Braingame Brain: executive function training	Trend toward improvement in working memory and cognitive flexibility	Trend toward improvement in ADHD behavior	No significant change	The Netherlands
Miyajima et al. (130)	14 (7^*^/7)	ASD	Global cognitive function, executive function, attention sustain ability, social functioning	Frontal/executive program: cognitive flexibility, working memory and planning training	Improvement in global cognition (working memory, verbal fluency, planning and problem solving)	Improvement in social functioning	-	Japan

(Cognitive training program participants

* */comparative group or treatment as usual group)*. *(-) Not assessed domain*.

**Table 8:** RCT studies on CR effect on anorexia nervosa.

**References**	**Participants (N)**	**Participants characteristic**	**Purpose of cognitive remediation**	**Assessed domains**	**Types of cognitive remediation training program**	**Neurocognitive outcomes**	**Body weight**	**Anorexia nervosa related symptoms (effect size)**	**Country**
Lock et al. (78)	46 (23^*^/23)	Anorexia nervosa	Reduction of attrition	Cognitive function Body weight Anorexia nervosa related symptoms	Cognitive remediation program for Anorexia nervosa	Significant effect was found on set-shifting relative to comparative group in short-term, but there was no significant difference between groups in follow-up	No significant difference between groups	No significant change on disorder related symptoms	USA
Brockmeyer et al. (137)	25 (11^*^/14)	Anorexia nervosa	To investigate the feasibility and efficacy of specifically tailored cognitive remediation training for anorexia nervosa	Cognitive function	Computerized training based on cognitive flexibility	Better performance on cognitive set-shifting relative to comparative group (*d* = .62)	-	Both groups showed high treatment acceptance	Germany
Steinglass et al. (138)	30 (15^*^/15)	Anorexia nervosa	To improve eating behavior during weight restoration	Caloric intake	Cognitive remediation program for Anorexia nervosa	-	While caloric intake of comparative group increased, caloric intake of cognitive remediation group decreased	-	USA
Dingemans et al. (79)	75 (38^*^/37)	Anorexia nervosa	To investigate the efficacy of cognitive remediation treatment on improving treatment effect of chronical or severe anorexia nervosa	Cognitive function Body Mass Index(BMI) Anorexia nervosa psychopathology Depressive symptom Anxiety Quality of life	Cognitive remediation program for Anorexia nervosa	No significant difference between groups	No significant difference between groups	Improved quality of life of anorexia nervosa relative to control group (*d* = 1.36)	The Netherlands

(Cognitive training program participants

* */comparative group or treatment as usual group)*. *(-) Not assessed domain*.

Previous studies have investigated the therapeutic efficacy of both pharmacotherapy and CR. Recently, however, studies have begun to combine these two therapies to improve cognitive function. The main purpose of these studies are to see if there are any synergistic effects when these two therapies are combined. Based on the results of Hampson et al. (152), where rats demonstrated a better performance when a drug was combined with behavioral strategies, researchers are considering that a combination of pharmacotherapy and CR could be more effective than a single therapy (153). The effects of providing a combination of CR and pharmacotherapy in schizophrenia have been reported in three studies so far. In these studies, D'souza et al. (154) reported that no significant improvements in cognitive function were shown when D-serine, a glutamatergic drug, was administered in combination with CR. In a subsequent study by Michalopoulou et al. (155) that combined modafinil with cognitive rehabilitation, there was no significant change in cognitive function compared to the control group. Alternatively, Cain et al. (156) showed improvement in auditory discrimination task performance when the glutamatergic drug D-cycloserine was combined with CR, as well as improvement in negative symptoms. Few studies have been conducted; therefore, it is too early to determine, based on existing research results alone, whether combining CR therapy with pharmacotherapy has synergistic effects. Since this is a subject that has been studied relatively recently, it is expected that research on this topic will be active in the near future.

Recently, studies have been conducted to design a method to improve the therapeutic effects of CR, the effectiveness of which has mainly been proven in schizophrenia. Although the effects were verified through several studies, the 37% drop-out rate was a limiting factor when CR was performed in real world settings (157). In addition, low motivation may hinder participation in psychosocial treatment and negatively affect treatment outcomes (158, 159). Considering that adherence and participant engagement in the program is related to future functioning, a way to combine CR and motivational enhancement interviewing techniques (ME) to solve these limitations has also been studied recently. Fiszdon et al. (160) conducted a study to improve the effectiveness of CR by improving intrinsic motivation. The results showed that combining CR with ME did not improve cognitive function compared to the CR only group. However, internal motivation and the attendance rates of training sessions were higher in the ME group. These findings suggest that intrinsic motivation is an important factor in providing CR for schizophrenia. More research is needed to improve the effectiveness of these treatments so that patients can better benefit from CR.

In recent years, although not covered in this study, the digital transformation of CR (e.g., internet-based CR, mobile application for CR, therapist-free CR) has been accelerating. This new shift, driven by the widespread availability of digital technology has been taking place throughout the psychological treatment as well as CR (161). However, as Heeren (162) pointed out, most digital transformation of psychological treatment has been neither theory-driven nor evidence-based. The number of quality studies related to digital transformation of CR is still lacking. Nonetheless, when considering the shift from paper and pencil training to computer-based training, it is expected that the digital transformation of CR can bring many benefits. For future CR, it is important to investigate the quality, effectiveness, adherence of digital CR interventions. More quality studies are urgently needed in this field.

Several limitations of the current review should be noted. First, the potential publication bias could not be eliminated. Although all of the studies we reviewed did not demonstrate positive results, this review still has potential publication bias because only published studies were targeted. Second, it should be noted that CR studies of other psychiatric disorders, except schizophrenia, were too premature to draw conclusions. Further studies should elucidate the effects of CR on cognitive functioning, symptoms, and other psychosocial functions in various psychiatric disorders.

## Author contributions

EK and K-HC designed the review, EK conducted a literature search and wrote the first draft. EK, Y-CB, HO, J-SL, W-HL, and K-HC revised a subsequent manuscript.

### Conflict of interest statement

The authors declare that the research was conducted in the absence of any commercial or financial relationships that could be construed as a potential conflict of interest.
